# Treatment with eFT-508 increases chemosensitivity in breast cancer cells by modulating the tumor microenvironment

**DOI:** 10.1186/s12967-022-03474-9

**Published:** 2022-06-18

**Authors:** Zhao-ying Yang, Cheng-wei Jiang, Wen-long Zhang, Guang Sun

**Affiliations:** 1 Department of Breast Surgery, China-Japan Union Hospital of Jilin University, No.126, Xiantai Street, Changchun, 130033 Jilin China; 2 Department of Pathology, China-Japan Union Hospital of Jilin University, Changchun, 130033 Jilin China; 3 Department of Hematology and Oncology, China-Japan Union Hospital of Jilin University, Changchun, 130033 Jilin China

**Keywords:** Triple-negative breast cancer, TNBC, Programmed death-ligand 1, PD-L1, eFT-508, Adriamycin

## Abstract

**Background:**

Patients with triple-negative breast cancer (TNBC) are better responders to neoadjuvant chemotherapy; however, they are poor in the durability of response with decreased overall and progression-free survival.

**Methods:**

Given that significant improvements have been reported with PD-L1-PD-1 blockade in different cancers, we evaluated the in vitro and in vivo effectiveness of Tomivosertib (eFT-508), an anthracycline, adriamycin, and MNK1/2 inhibitor, which has been previously shown to inhibit translation of PD-L1 in mice model of liver cancer, alone or in combination using BC cell lines and an orthotopic xenograft mice model using the TNBC cell line MDA-MB-231.

**Results:**

Within the context of The Cancer Genome Atlas (TCGA) dataset, expression of *CD274* mRNA, which encodes programmed death-ligand 1 (PD-L1), was found to be significantly overexpressed in TNBC patients compared to patients with HER2 + or luminal breast cancer (BC). Even within TNBC sub-types, *CD274* expression was significantly higher in the immune modulatory subtype (TNBC-IM).

BC cells exhibited high IC_50_ = 0.85 ± 0.07 nM with Adriamycin and significantly lower IC_50_ = 0.23 ± 0.04 nM with eFT-508 (*P* < 0.01). Combination treatment showed in vitro synergism on chemosensitivity. Combination therapy also exhibited a synergistic effect on inhibition of tumor growth and lung colonization in vivo. Mass cytometry-based evaluation of the tumor microenvironment revealed significant attenuation of both PD-L1 and PD-L2 following mono- or combination therapy with eFT-508.

**Conclusions:**

Treatment with eFT-508 restored effector and cytotoxic function of tumor-infiltrating CD8 + T cells in mice. The remarkable efficacy observed both in vitro and in vivo, and clinical synergism with adriamycin, highlights the potential of eFT-508 as an alternative, yet more efficacious, therapeutic option for patients with TNBC.

**Supplementary Information:**

The online version contains supplementary material available at 10.1186/s12967-022-03474-9.

## Background

Breast cancer (BC) is still the leading cause of women's fatality [1, 2]. Among them, triple-negative breast cancer (TNBC) is a subtype of BC, in which tumors do not express HER2/neu, estrogen receptor (ER), or progesterone receptor [3, 4]. TNBC accounts for about 20% of all BC cases [3, 4]. TNBC is a low-differentiated, high-grade invasive tumor with a high tendency of metastasis and poor prognosis [3, 5]. With the understanding of TNBC and the improvement of treatment methods, the survival rate of patients with BC has been improved, but the mortality caused by chemotherapy resistance and postoperative tumor metastasis is still rising [6, 7]. Therefore, it is still necessary to find more pathogenic mechanisms in a clinical setting to provide more effective treatment entry points for these patients [8].

TNBCs are highly heterogeneous and are further sub-divided into six sub-types based on the gene expression profiles [4]. The immunomodulatory sub-type (TNBC-IM) is characterized by enrichment of genes associated with antigen cross-presentation, immune, and cytokine signaling [4]. In addition, TILs often lose function due to the expression of immune checkpoint receptors, including the expression of programmed cell death protein 1 (PD-1) [9]. PD-1 binds to its two ligands, PD-L1 and PD-L2, which are expressed on the surface of tumor cells [10]. The breast tumors express PD-L1, and this expression is related to the negative receptor state [10, 11]. In addition, studies have found that the treatment effect of patients with TNBC is significantly improved using anti-PD-1/PD-L1 single drug treatment (29,424,936). However, their exact role in the pathogenesis of TNBC is still unclear.

The cells in primary tumors are mainly epithelial in nature, which are tightly bound and cannot migrate to secondary sites. They undergo phenotypic changes (lower expression of epithelial cell markers and higher expression of mesenchymal cell markers) and functional changes (increased migration and invasiveness) to make cancer cells migrate to secondary sites. This process is called epithelial to mesenchymal transition (EMT) [12, 13]. Anthracycline class drugs, such as Doxorubicin (adriamycin) are used in chemotherapy of early and metastatic BC in combination with other drugs [14]. Animal model studies have shown that the antitumor effect of anthracyclines in fully immunized mice is better than that in immunodeficient mice, indicating that the activation of this adaptive immune response depends on the role of the immune system. Anthracyclines, a class of chemotherapeutic drugs, can induce tumor cell death, activate the immune system to fight against dead cell antigens, and endow dead tumor cells with immunogenicity. However, they have low efficacy and high toxicity [15]. The inhibitors of protein translation are increasingly being tested in clinical trials to treat cancers and have shown promising initial results [16]. Mitogen-activated protein kinase (MAPK)-interacting kinase 1 and 2 (MNK1/2) phosphorylates eIF4E at Serine 209 residue. Phosphorylation of eIF4E initiates a cascade of events, leading to the initiation of translation [16]. A recently developed MNK1/2 inhibitor, Tomivosertib or eFT-508 [16], has been shown to inhibit the expression of PD-L1 and slow down tumor progression in the mouse model of liver cancer [17], but the efficacy in TNBC is still unknown.

Therefore, in the present study, we tested the in vitro and in vivo effectiveness of adriamycin and eFT-508 alone or in combination using BC cell lines and an orthotopic xenograft mice model using the TNBC cell line MDA-MB-231. EFT-508 alone showed significant efficacy both in vitro and in vivo and showed a synergistic effect with adriamycin. Thus, it acted as a viable alternative and a more effective treatment option for patients with TNBC.

## Methods

### Cell culture and treatment

The human BC cell lines, MDA-MB-231, MDA-MB-468, MCF7, T47D, and SUM149 (American Type Culture Collection, Manassas, USA) were cultured in Dulbecco’s Modified Eagle Medium (DMEM, Thermo Fisher Scientific, USA). The medium was supplemented with 10% fetal bovine serum (FBS, Invitrogen, Carlsbad, CA, USA) and 1% penicillin–streptomycin solution. All cell lines were incubated in 5% CO_2_ at 37 °C where indicated cells were treated with indicated doses of adriamycin (Doxorubicin) and/or Tomivosertib (eFT-508) (Selleckchem) for 24 h.

### Polysome profiling

Polysome profiling was done following a previously described protocol [18]. Briefly, cells were treated with 50 µg/mL with cycloheximide (Sigma-Aldrich, St. Louis, USA) for 30 min at 37 °C and then washed with cycloheximide-supplemented cold PBS. Cells were lysed, and post-nuclear extracts were layered on 10–50% sucrose gradients and centrifuged at 100,000*g* for 4 h. Gradient fractionation was performed using the UA-6 UV detector (Teledyne ISCO, US) based on 254 nm absorption over time.

### Puromycin end-labeling assay

After the indicated treatment regimen, puromycin (C58-58–2, Sigma Aldrich) was added at a final concentration of 1 μM (100-fold dilution) for 12 h, and the cells were cultured for an additional 30 min before harvesting and cell lysate preparation. Cell lysates were subjected to immunoblot analysis. Blots were probed with an anti-puromycin antibody (Kerafast) to assay relative puromycin incorporation. Finally, blots were stained with Coomassie to confirm equal loading across different samples.

### Immunoblot analysis

Normal cells and BCs were plated in 6-well plates at a density of 3.0 × 10^5^ cells/well and cultured in CCSCs medium. After 24 h, cells were transferred to serum-free medium and incubated for 18 h. After the cells were treated according to the established groups, the cells were collected. Next, we added cell lysis buffer (50 mm tris–HCl, pH 7.5, 150 mm NaCl, 1 mm sodium-EDTA, 1 mm EDTA, 1% (v/v) Triton x-100, 2.5 mM sodium pyrophosphate, 1 mM β-glycerophosphate, 1 mM Na_3_VO_4_, 1 mM NaF, 1 μg/mL leupeptin, and 1 mM PMSF). The protein concentration was determined by the BIORAD reagent, and the loading volume of protein was calculated. Protein bands were separated based on molecular weights via 10% (w/v) SDS-PAGE (electrophoresis conditions: concentration gel 100 V, 25 min; separation gel 200 V, 40–50 min), and then transferred to PVDF membrane (transfer condition: 100 V, 2 h). The membrane was sealed with 5% (w/v) skimmed milk powder at room temperature for 1.5–2 h, followed by the addition of corresponding primary antibody and incubate at 4 °C overnight. Finally, the secondary antibody was incubated at room temperature for 1.5–2 h, and the protein image was obtained using a gel imager. The following primary antibodies were used: anti-P-eIF4E (ab1126), eIF4E (ab238519), and GAPDH (ab181603), all antibodies were purchased from Abcam China and used at 1:1000 dilution. GAPDH was used as the loading control.

### Quantification of cytokines

We added 200 μL of assay buffer to the cells in the wells of the experimental titration plate and then placed on a shaking table for 10 min. The assay buffer was removed with a vacuum pump; 25 μL of assay buffer was added into the sample hole and appropriate matrix solution was added into the standard hole and quality control hole of back measuring hole. Finally, 25 μL of the sample was added into the appropriate hole, shook, and 25 μL of the microspheres were mixed into each hole. The board was closed with special plastic film and shook at 4 °C for 18–20 h. The residual liquid was gently removed with a vacuum pump. Next, 200 μL of the wave buffer was added to each hole for cleaning, followed by the addition of 25 μL of secondary antibody into each hole. The plate was incubated in a shaker for 2 h, then 25 µL of streptavidin phycoerythrin was added into the well containing the second antibody. The cells were incubated on shaking table for 30 min at room temperature, followed by the addition of 150 µL of sheath solution to all holes. The expressions of TNFα, IL-10, IL-8, CXCL-10, and IL-6 were measured by Luminex (Millipore) in plasma samples obtained from mice in different experimental groups.

### Cell isolation, culture, and treatment

Positive selection was used to isolate CD8 + T cells (10^4^ cells/hole) from tumors using magnetic-activated cell sorting using CD3 and CD8 microbeads (Miltenyi Biotec). The cells were cultured in 6-well plates coated with OKT3 (Ortho Biotech; 1 μg/mL) and anti-CD28 (clone CD28.2, Becton Dickinson; 1 μg/mL) antibodies. The cells were cultured in DMEM medium without double antibody for 4 days, and the supernatant was aspirated, and the PCR method and culture method were used to detect whether there was mycoplasma contamination. The cells in the culture flask with good growth condition and cell fusion degree of 75–80% were washed twice with PBS buffer and digested with trypsin. The degree of cell digestion was observed under a microscope. When the cells became round, and the intercellular space became larger, an appropriate amount of 10% (v/v) FBS 1640 medium was added to stop the digestion. The cells were gently blown down using a blow tube and transferred into a 15 mL centrifuge tube. After centrifugation at 800 rpm for 5 min, the supernatant was discarded and passaged at a ratio of 1:2.

### Determination of cell viability by MTT assay

The cells of each group planted in the 96-well plate were taken, and after culturing for 48 h, 20 μL of MTT solution was added to the cells, and the incubation was continued for 4 h. Remove the supernatant solution with a pipette, add 150 μL of DMSO to each well, observe the dissolution of the crystals, and measure the absorbance (A) value of each well at 490 nm. A larger A value indicates higher cell viability.

### Spontaneous xenograft metastasis model

All animal experiments were approved by the Laboratory Animal Welfare and Ethics Committee of the Third Military Medical University. MDA-MB-231 cells (0.5 × 10^6^) were trypsinized, washed with PBS, and resuspended in a 1:1 solution of PBS and Matrigel (phenol red-free and reduced growth factors, BD Biosciences) and injected into the 4th mammary fat pad of 8-week old female BALB/c mice (n = 20). Tumor volume was measured every day using a caliper. When the tumors reached 50 mm^3^ in size (around day 7), the mice were randomly divided into four experimental groups (n = 5/group)—vehicle (DMSO), adriamycin (3 mg/kg/day), eFT-508 (1 mg/kg/day), and eFT-508 (1 mg/kg/day) + adriamycin (3 mg/kg/day)—all injections were intratumoral. Bodyweight, tumor growth rate, and survival were analyzed for each group. The mice were sacrificed either when tumor volume reached 3000 mm^3^ or at the end of 8 weeks, whichever was earlier. The tumors and lungs were surgically removed, weighed, fixed in 4% polyformaldehyde, and stored at − 80 °C.

### Hematoxylin & eosin (H&E) and Immunohistochemistry (IHC) staining

Next, 4 µm sections were either processed for H&E staining using routine methodologies or IHC. The sections were dewaxed using xylene I and II for 10 min, respectively. A cover glass was prepared in advance, covered with water, 100% (I, II), 90%, 80%, and 70% alcohol for 5 min, respectively, and rinsed with tap water for 15 min; dyed with hematoxylin for 5 min. The dyeing time could be appropriately increased or reduced. After differentiation with 5% acetic acid for 1 min, the sections were rinsed with running water, followed by the addition of acetic acid with a pipette, and the tissue was covered on the slide. After differentiation, the color became light and blue; Blue returning: Blue returning liquid, which is not available in the laboratory, can also be used; the sections were stained with Eosin for 1 min, dehydrated using 70%, 80%, 90%, and 100% alcohol for 10 s and xylene for 1 min. The sections were dried naturally in the fume hood and then sealed for about 5 min. Next, a drop of neutral gum was dropped to seal the film, and a straw was used to drop a very small drop; the tissue was covered after pressing the film to avoid bubbles in the middle. For IHC, sections were treated for antigen retrieval and incubated with primary antibodies (P-eIF4E, 1:200; Cell Signaling Technology) overnight at 4 °C. The images were obtained using an Olympus light microscope. We randomly selected three visual fields and analyzed three slices per sample at 20×magnification.

### Mass cytometry (CyTOF) staining

Cells were stained with cisplatin (DVS-Sciences) to identify live cells. Then chemoresistant cell lines were incubated with 19 metal-tagged anti-PD-L1 antibodies (Biolegend, 124,314), followed by an intercalator (DVS Sciences). Staining was done following guidelines of the Maxpar cell staining protocol (Fluidigm Corporation) and then incubated at 4 °C for 30 min [17]. CyTOF-II mass cytometer (Fluidigm) was used for sample runs. Post-run, the EQ Four Element Calibration Beads (EQ Beads, 201,078, Fluidigm) were used to normalize the signal. Events were initially gated for viability using Ir191 vs. Ir193, and double-positive cells were used in downstream analyses to identify different sub-populations post-dynamic downsampling.

### Mixed lymphocyte reaction (MLR) assay

Responders CD3 + CD8 + T cells, sorted from tumor-infiltrating immune cells, were stained with CFSE (Thermo Fisher Scientific) and seeded into 96-well plate format in RPMI. Also, 10% AB serum either alone or in combination with irradiated (40 Gy) allogeneic PBMCs (stimulators) at three different ratios—1:1, 2:1, and 5:1. CD14^+^ antigen was present in all wells. T cell proliferation was calculated by analyzing the CFSE dilution of the responders after 72 h.

### Effector T cell functional assay

Sorter tumor-infiltrating CD3 + CD8 + cells were cultured in medium ± PMA (20 ng/mL; Sigma-Aldrich, St. Louis, MO) and Ionomycin (1 µg/mL; Sigma-Aldrich). Luminex (Millipore) was used to quantify the levels of indicated cytokines in the supernatants collected before or after 3 days of PMA/Ionomycin stimulation.

### Data mining

Transcriptomic analyses of *CD274* expression data in invasive breast carcinoma from The Cancer Genome Atlas (TCGA) were performed using the interactive web resource UALCAN (http://ualcan.path.uab.edu; Accession date: November 8, 2020) [19]. Survival probability based on low and high expression of *CD274* was computed by Kaplan–Meier curve analysis and Log-rank text using USALCAN interactive portal.

### Statistical analysis

Quantitative data were presented as mean ± standard deviation (SD). Statistical analysis between groups was performed using Student’s *t*-test (two-group comparison) or ANOVA (multiple group comparisons). Kaplan–Meier curves were computed to analyze survival rates, and Log-rank (Mantel-Cox) test was used to evaluate statistical significance. A *P*-value < 0.05 was considered to be statistically significant.

## Results

### Expression of mRNA encoding PD-L1 is higher in TNBC-IM subtypes

**Fig. 1:**
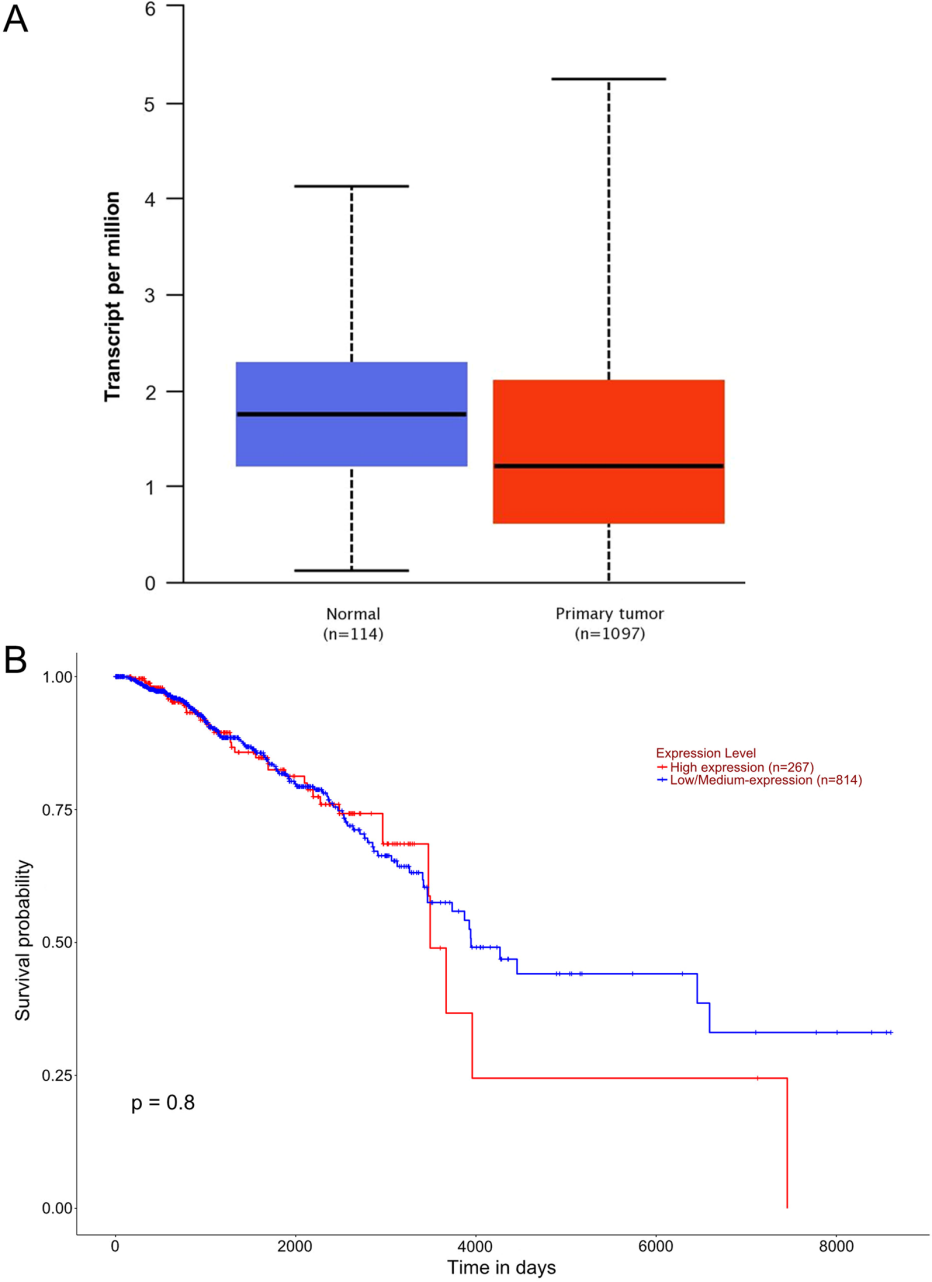
Expression of *CD-274* transcript, which encodes PD-L1, is not correlated to the overall survival in patients with BC. **A** Boxplots showing the expression of *CD274* mRNA in unpaired BC tissues and normal breast tissues (UALCAN) (*P* = 0.049). **B** Kaplan–Meier plots revealed no association between the expression of *CD274* with the overall survival of patients with BC (*P* = 0.8)

We initially determined *CD274* (encodes PD-L1) mRNA expression within the TCGA dataset in invasive breast carcinoma (BRCA). The expression of *CD274* mRNA was slightly significantly lower in primary tumor tissues compared to the normal tissues (Fig. 1A; *P* = 0.049305). However, there was no correlation between *CD274* expression and the overall survival in patients with BC (Fig. 1B; *P* = 0.8). There was no correlation between *CD274* expression and disease stage (Additional file 1: S1A) and race (Additional file 1: Figure S1B).

**Fig. 2:**
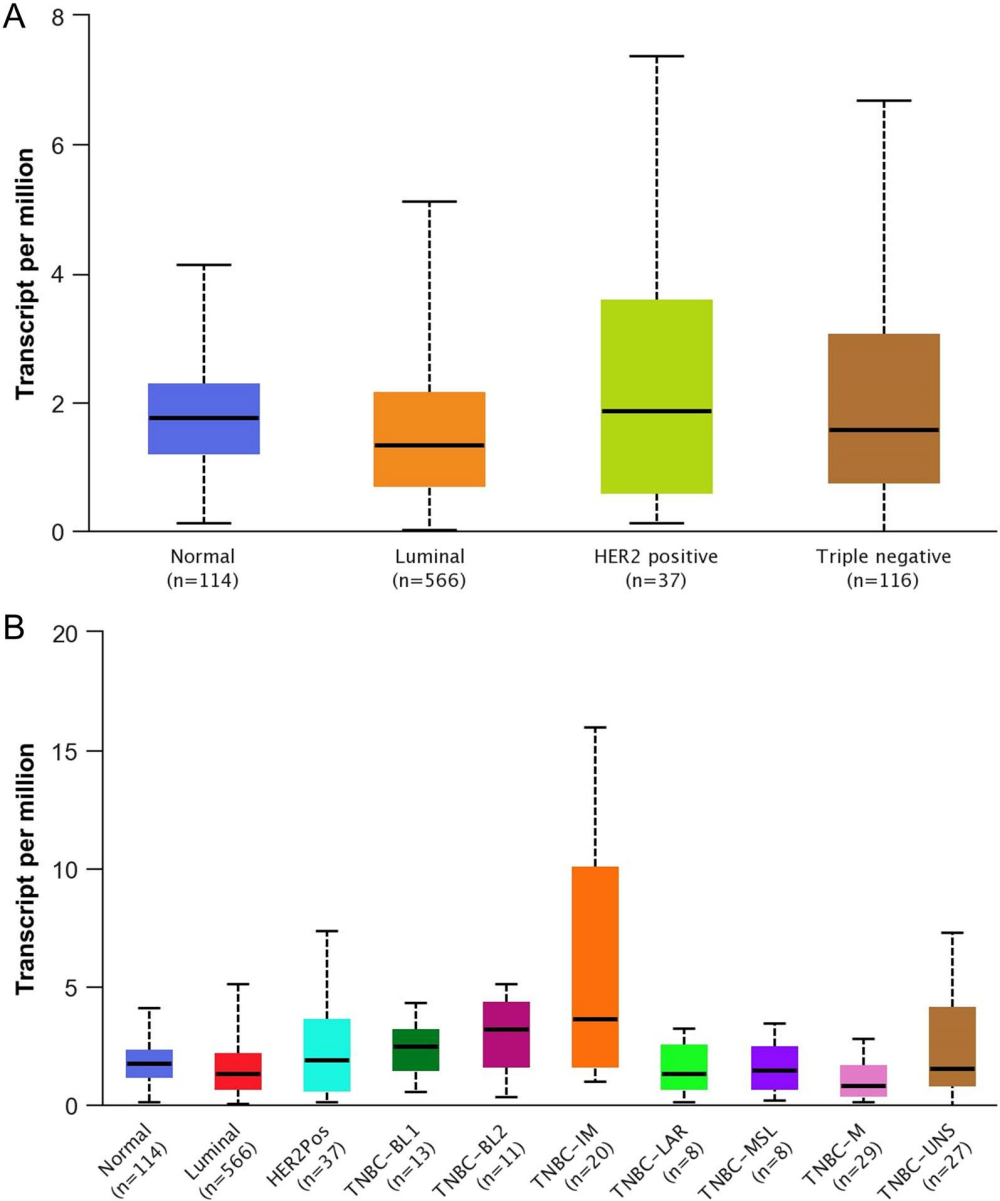
Expression of CD274 transcript is significantly higher in TNBC-IM. **A** Boxplots showing the expression of *CD274* mRNA in normal breast tissue and luminal, HER2 + , and triple-negative BC (UALCAN). **B** Boxplots showing the expression of *CD274* mRNA in normal breast tissue and BC classes, including sub-types of TNBC (UALCAN)

Next, we evaluated *CD274* expression in BRCA based on BC subclasses, luminal, HER2 + , and TNBC. The expression of *CD274* was higher compared to both normal (*P* = 0.0101171) and luminal (*P* = 0.023656), but not HER2 + (*P* = 0.103021) (Fig. 2A). Then we determined *CD274* expression within the different subtypes of TNBC. The expression of *CD274* was statistically significant different in TNBC-IM compared to normal (*P* = 0.0138987), luminal (*P* = 0.016004), HER2 + (*P* = 0.020079), TNBC-BL1 (*P* = 0.025676), TNBC-LAR (*P* = 0.010937), TNBC-MSL (*P* = 0.0119235), and TNBC-UNS (*P* = 0.030065) (Fig. 2B). Thus, PD-L1 mRNA expression had a significant correlation with the receptor-negative status [13, 14], especially to the TNBC-IM subtype.

### *Combination treatment with eFT-508 and adriamycin exhibits a synergistic effect on *in vitro* chemosensitivity of BC cell lines*

**Fig. 3:**
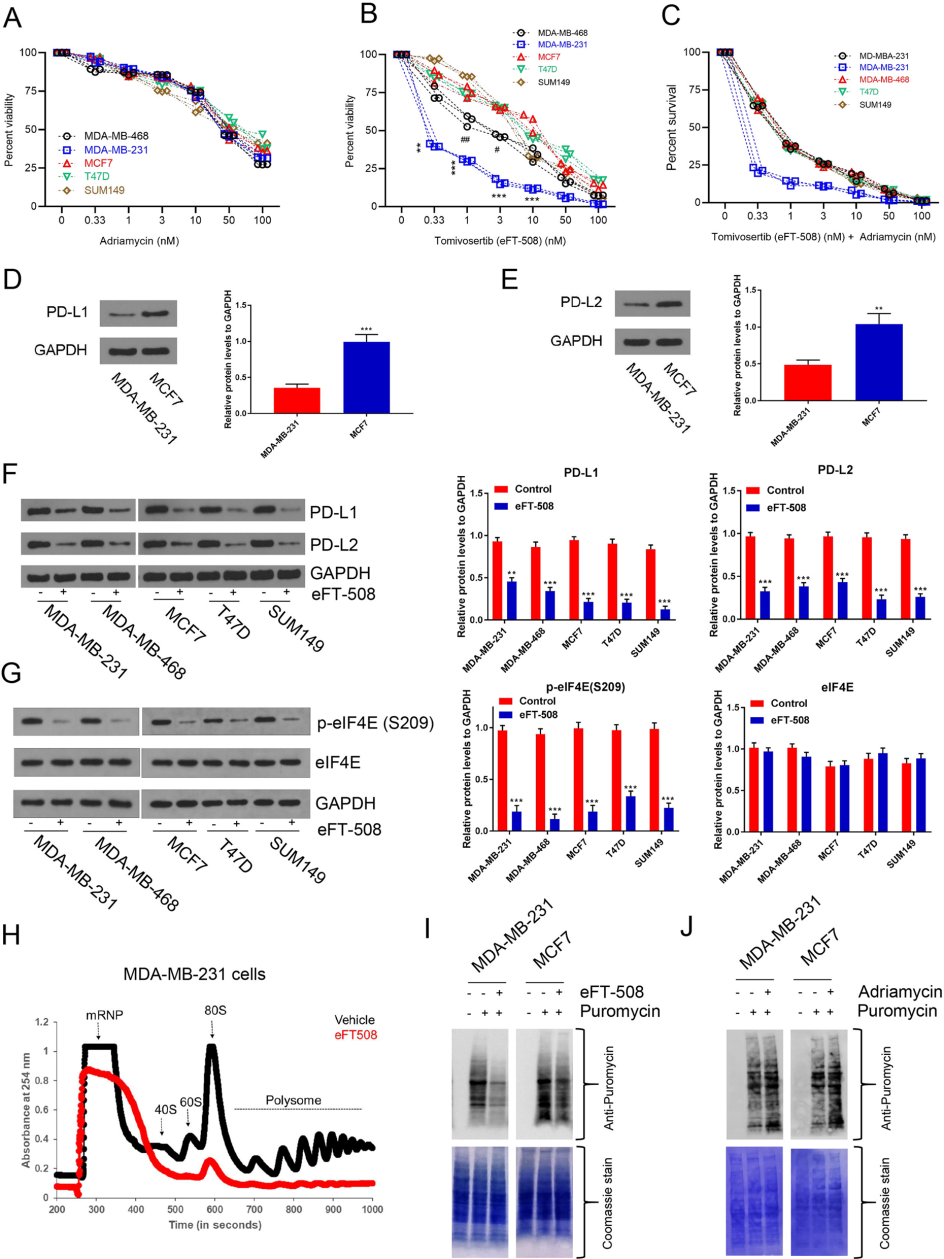
Synergistic effect of eFT-508 and adriamycin on the in vitro chemosensitivity of BC cell lines. **A** BC cell lines are chemoresistant to adriamycin with high values of IC_50_. Exponentially growing cultures of BC cell lines, MDA-MB-231, MDA-MB-468, MCF7, T47D, and SUM149, were treated with increasing concentrations of adriamycin for 24 h. Cell viability was evaluated using MTT assay, and percent viability after 24 h were plotted. No significant difference was observed between the cell lines for any of the concentrations of adriamycin tested. The selection criteria of drug dilution were scalar dilution factor 3 and then factor 2. **B** Differential sensitivity of BC cell lines to Tomivosertib (eFT-508). Same as **A** but was treated with increasing concentrations of eFT-508. All cell lines tested had more chemosensitivity to eFT-508 compared to the same concentration of adriamycin. MDA-MB-468 and MDA-MB-231 were more chemosensitive compared to the other three cell lines. The selection criteria of drug dilution involved scalar dilution factor 3 and then factor 2. ^#^*P* < 0.05; ^##^*P* < 0.01; **P = 0.001; ***P < 0.001. **C** Adriamycin, in combination with eFT-508, showed a higher in vitro therapeutic index. Same as **A** but instead treated with both adriamycin and eFT-508. Combination therapy resulted in significantly less IC_50_ in all cell lines compared to monotherapy. The IC_50_ in the MDA-MB-468 cells was significantly lower compared to the other four cell lines. The selection criteria of drug dilution included scalar dilution factor 3 and then factor 2. Data in **A–C** are represented as staggered plots, with each point representing an experimental replicate (n = 3). **D** Western blot was used to detect the expression of PD-L1 in BC cell lines. **E** Western blot was used to detect the expression of PD-L2 in BC cell lines. **F** Treatment with eFT-508 resulted in the inhibition of all five cell lines, and Western blot was used to detect the expression of PD-L1 and PD-L2 in cell lines. **G** Treatment with eFT-508 resulted in the suppression of MNK1/2-mediated phosphorylation of eIF4E in all five cell lines. Immunoblot analysis of phosphorylated eIF4E (S209) and total eIF4E in the cell lines treated with eFT-508. Shown are representative blots from three biological replicates. GAPDH was used as a loading control. **H** Treatment with eFT-508 resulted in the inhibition of global translation. Representative polysome profile obtained from the post-nuclear extract of vehicle or eFT-508 treated MDA-MB-231 cells. Treatment with eFT-508 resulted in the lower 80S and polysome peaks. Similar results were obtained in the other four cell lines as well (*data not shown*). **I** Results in **E** were additionally verified using the SUrface SEnsing of Translation (SUnSET) technique to assess changes in global translation. Untreated or eFT-508-treated MCF7 and MDA-MB-231 cells were labeled with puromycin for 15 min. Shown are immunoblot analyses of lysates made from these cells using an anti-puromycin antibody (*top*). The membrane was subsequently stained with Coomassie Blue to ensure equal protein loading across lanes. Shown are representative blots from three experiments. **J** Treatment with adriamycin had no noticeable effect on global translation. Untreated or adriamycin-treated MCF7 and MDA-MB-231 cells were labeled with puromycin for 15 min. Shown are immunoblot analyses of lysates made from these cells using an anti-puromycin antibody (*top*). The membrane was subsequently stained with Coomassie Blue to ensure equal protein loading across lanes. Shown are representative blots from three experiments

**Fig. 4:**
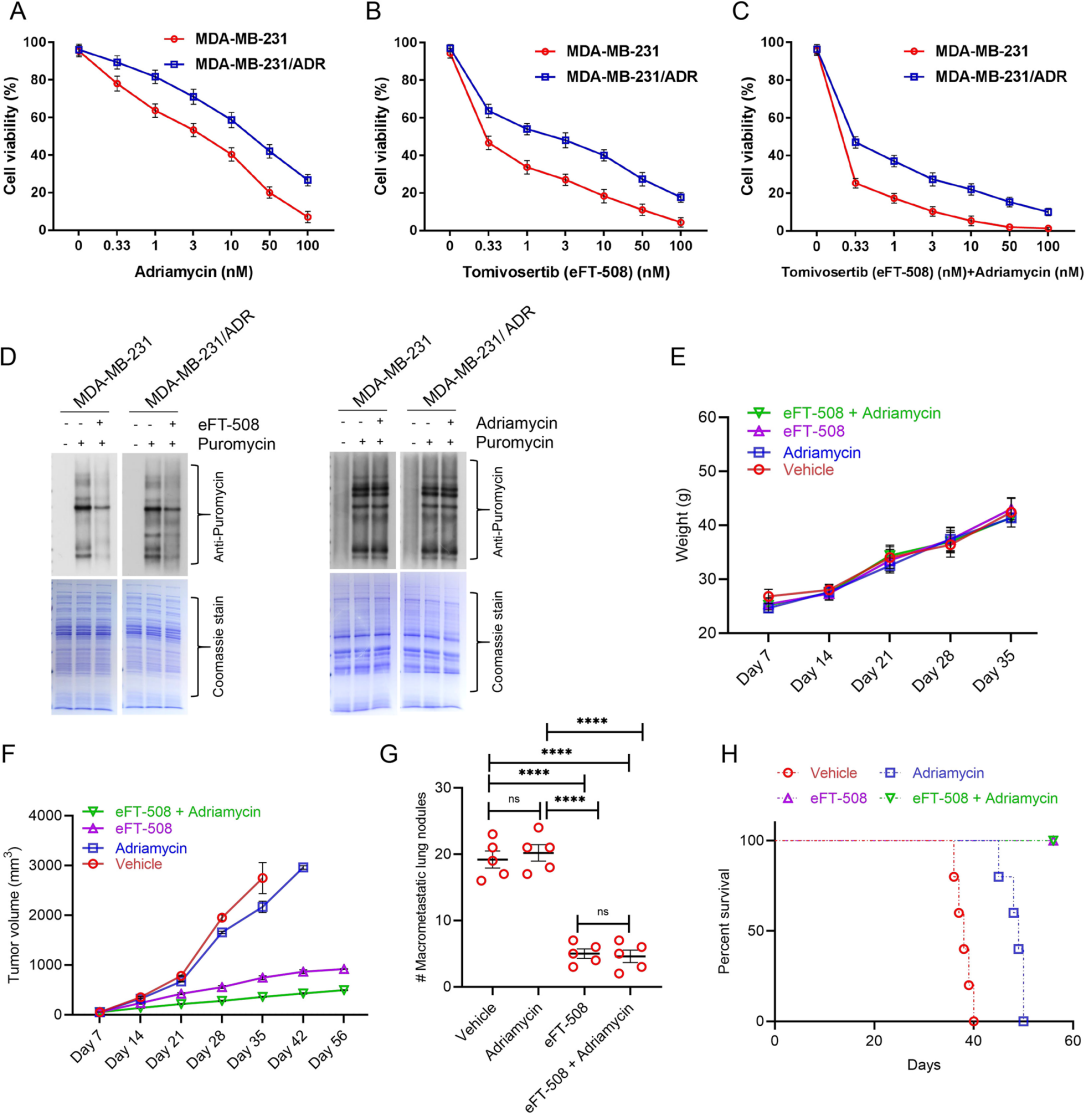
Synergistic effect of eFT-508 and adriamycin on in vivo chemosensitivity of breast tumor xenografts. **A** Cell viability (%) of MDA-MB-231, MDA-MB-231/ADR cell lines treated with Adriamycin. **B** Cell viability (%) of MDA-MB-231, MDA-MB-231/ADR cell lines treated with Tomivosertib (eFT-508). **C** Cell viability (%) of MDA-MB-231, MDA-MB-231/ADR cell lines treated with Adriamycin and Tomivosertib (eFT-508). **D** Total protein was detected by SDS-PAGE after MDA-MB-231, MDA-MB-231/ADR cell lines treated with Adriamycin, Tomivosertib, Adriamycin and Tomivosertib (eFT-508). **E**–**H** MDA-MB-231 cells were injected into the mammary fat pad of BALB/c mice (n = 20). When tumors reached a volume of 50 mm^3^, mice were randomly assigned to four groups (n = 5/group) – vehicle, adriamycin, eFT-508, and adriamycin + eFT-508 via intratumoral administration. Tumor and lung tissues were harvested either at the end of 8 weeks or when tumor volume reached 3000 mm^3^ or when mice died, whichever was earlier. **E** Changes in body weight of mice in the different experimental groups over 5 weeks. **F** Tumor growth rate in the different experimental groups. All mice in the vehicle group died by week 6, whereas mice in the adriamycin group died by week 7. **G** Quantification of macrometastatic lung nodules in mice from the different experimental groups. For **E**–**G**, data are represented as mean ± standard deviation; ****P < 0.0001, ns – not significant (*P* > 0.05) (n = 5 mice each group). **H** Survival analysis of mice in different experimental groups was assessed by Kaplan–Meier plots. Survival in eFT-508 monotherapy and combination therapy groups was significantly higher compared to either vehicle or Adriamycin groups (*P* < 0.0001 in each case, log-rank test)

Since PD-L1 mRNA expression was significantly higher in TNBC patient samples, we next evaluated if cancer cell lines representing different subtypes had different chemosensitivity to adriamycin and eFT-508 alone and in combination. Luminal cell lines MCF7 and T47D, basal-like, EGFR + SUM149, and TNBC cell lines MDA-MB-231 and MDA-MB-468 cells were used. The exponentially growing cultures of these cell lines were treated with increasing concentrations of adriamycin for 24 h. All cell lines were chemoresistant with a high IC_50_ ranging from 48.3 to 49.2 nM (Fig. 3A). However, when these cells were treated with increasing concentration of eFT-508, MDA-MB-231 (IC_50_ = 0.31 ± 0.02 nM) and MDA-MB-468 (IC_50_ = 1.21 ± 0.01 nM) cell lines exhibited significantly higher chemosensitivity compared to MCF7 (IC_50_ = 11.38 ± 1.02 nM), T47D (IC_50_ = 10.19 ± 0.01 nM), or SUM149 (IC_50_ = 9.14 ± 0.14 nM) cell lines (Fig. 3B; *P* < 0.05 in each case). MDA-MB-231 cells were significantly more chemosensitive compared to MDA-MB-468 cells (Fig. 3B). Combination treatment with adriamycin and eFT-508 revealed higher in vitro chemosensitivity with significantly lower IC_50_ in all cell lines compared to either monotherapy (Fig. 3C). Again, the IC_50_ was significantly lower in MDA-MB-231 cells compared to each of the other four cell lines (Fig. 3C). Western blot results showed that PD-L1 (Proteintech, Mouse Monoclonal| Catalog number: 66248–1-Ig, Fig. 3D) and PD-L2 (Proteintech, Rabbit Polyclonal, Catalog number: 18251–1-AP, Fig. 3E) were highly expressed in BC cells. In addition, five cell lines were treated with eFT-508; the results of WB showed a decreased expression of PD-L1 and PD-L2 (Fig. 3F). Immunoblot analysis confirmed a robust decrease in P-eIF4E (S209) in each of the cell lines treated with eFT-508 without any change in total eIF4E expression (Fig. 3G). Polysome profiling indicated the inhibition of global translation following eFT-508 treatment in these cells, as evident by a decrease in both 80S and polysome peaks (Fig. 3H and *data not shown*). Polysome profiling results were further corroborated by puromycin labeling. Treatment with eFT-508 (Fig. 3I) but not Adriamycin (Fig. 3J) resulted in a robust decrease in puromycin labeling in both MDA-MB-231 and MCF7 cells. To determine if the observed results were due to clonality of the cell lines tested, we next determined the response of the parental MDA-MB-231 and multidrug resistant MDA-MB-231/ADR cells to adriamycin and eFT-508 treatment, alone or in combination. Indeed, the synergistic effect of adriamycin and eFT-508 was conserved in this model too (Fig. 4A–C). Again, the difference in chemosensitivity was not due to the differential effect of eFT-508 on translational inhibition as evidenced by puromycin labeling (Fig. 4D). Hence, the observed difference in chemosensitivity of the TNBC cell line MDA-MB-231 compared to the other cell lines tested was not due to the differential effect of eFT-508 on eIF4E phosphorylation and translational inhibition, indicating that the difference was instead dictated by the receptor negativity of the BC cell lines.

### *Combination treatment with eFT-508 and adriamycin exhibits a synergistic effect on *in vivo* chemosensitivity of breast tumor xenografts*

An orthotopic model of spontaneous metastasis was established by injecting MDA-MB-231 cells in the mammary fat pad of BALB/c mice. Once tumors reached a volume of 50 mm^3^, mice were randomly divided into four groups (n = 5/group)—vehicle (DMSO), adriamycin (3 mg/kg/day), eFT-508 (1 mg/kg/day), and eFT-508 (1 mg/kg/day) + adriamycin (3 mg/kg/day)—all injections were intratumoral. Tumors reached the size of 50 mm^3^ at approximately day 7 in all mice. Tumor growth, as well as metastatic dissemination, was followed for up to 8 weeks after the initial injection. Tissues were harvested either when mice were dead or when tumor volume reached 3000 mm^3^. All mice in the vehicle group died by the end of week 6. Hence body weights were measured for 5 weeks. No significant difference in body weight was observed among the animals from the different experimental groups (Fig. 4E). The tumor growth rate was significantly higher in the vehicle and adriamycin groups compared to eFT-508 or combination therapy groups (*P* < 0.0001 in each case). Combination therapy exhibited synergism in attenuating tumor growth rate compared to eFT-508 monotherapy (Fig. 4F; *P* < 0.05).

**Fig. 5:**
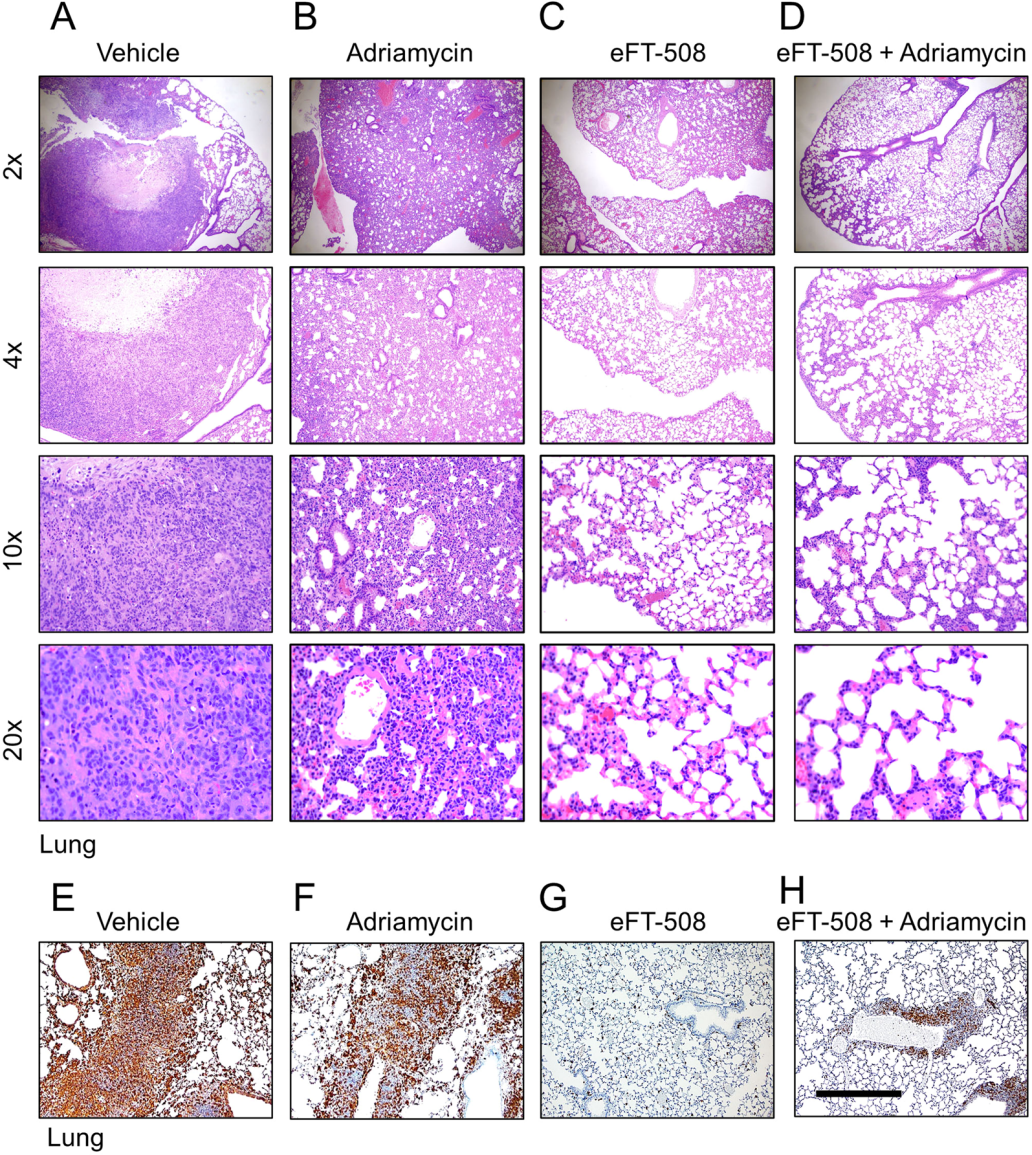
Treatment with eFT-508 inhibits lung metastasis. Lung tissues harvested from animals in the different experimental groups described in Fig. 4 were processed for H&E and IHC (P-eIF4E) staining. Shown are representative H&E staining at indicated magnifications (**A**–**D**) and IHC staining (**E**–**G**) from vehicle (**A**, **E**), Adriamycin (**B**, **F**), eFT-508 (**C**, **G**), and adriamycin + eFT-508 (**D**, **H**) experimental groups. Robust lung micrometastatic colonies were visible both in the vehicle (**A**) and Adriamycin (**B**) experimental groups. Lung metastasis was less in the eFT-508 group (**C**). Combination therapy with adriamycin and eFT-508 almost completely attenuated lung metastasis (**D**). Scale bar, 100 µm

After the animals were sacrificed, we counted the micrometastatized lung nodules and plotted them. Both eFT-508 monotherapy and combination therapy significantly reduced the incidence of macrometastatic lung nodules (Fig. 4G; *P* < 0.0001 compared to either vehicle or adriamycin group). No statistical difference was observed between eFT-508 monotherapy and combination groups (Fig. 4G; *P* > 0.05). Similarly, eFT-508 monotherapy or combination therapy significantly improved survival compared to vehicle or adriamycin groups (Fig. 4H). Thus, these results showed potent anti-tumorigenic and anti-metastatic effects of eFT-508 and revealed synergism with adriamycin only in the context of tumor growth rate. H&E staining of lung tissues (Fig. 5A–D). H&E staining showed that the combined drug group had fewer lung metastases than the single adriamycin group, which was consistent with the results in Fig. 4G. IHC staining confirmed the suppression of phosphorylation of eIF4E in the eFT-508 monotherapy (Fig. 5G) and combination (Fig. 5H) groups compared to vehicle (Fig. 5E) and adriamycin (Fig. 5F) groups.

### Combination therapy had a synergistic effect on the expression of PD-L1 and PD-L2 by the tumor cells

**Fig. 6:**
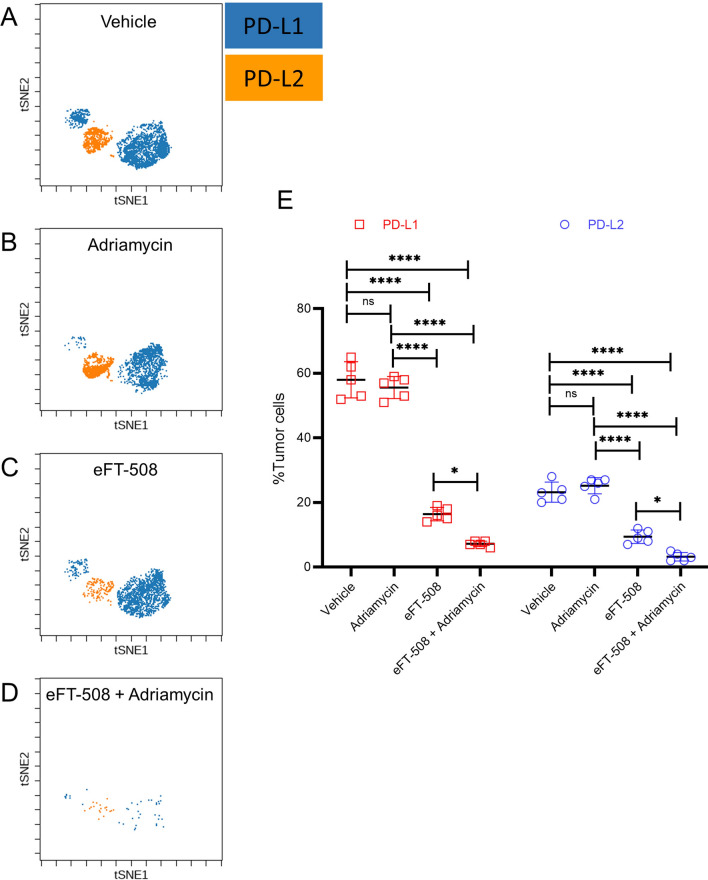
Treatment with eFT-508 leads to significant downregulation of PD-L1 and PD-L2. CyTOF (viSNE plots) analysis of PD-L1 and PD-L2 expression in tumor tissue obtained from xenograft mice (as described in Fig. 4) injected with either vehicle (**A**), adriamycin (**B**), eFT-508 (**C**), or adriamycin + eFT-508 (**D**). **E** Quantification of data shown in **A**–**D**. Data are represented as mean ± standard deviation; *P < 0.05, ****P < 0.0001, ns—not significant (*P* > 0.05) (n = 5 mice/group)

Next, we evaluated the tumor microenvironment for PD-L1 and PD-L2 expression using mass cytometry. Compared to tumors in the vehicle (Fig. 6A, E) or adriamycin treatment group (Fig. 6B, E), both eFT-508 monotherapy (Fig. 6C, E) and combination therapy (Fig. 6D, E ) significantly downregulated PD-L1 + and PD-L2 + breast tumor cells. Even though there was no significant difference in PD-L1 + and PD-L2 + breast tumor cells between vehicle and adriamycin groups, combination therapy with adriamycin and eFT-508 showed a synergistic effect compared to both adriamycin and eFT-508 monotherapy groups (Fig. 6C–E). These results indicated that treatment with eFT-508 was perhaps inducing immune modulation potentiating tumor cell clearance.

### Treatment with eFT-508 restores the functional activity of tumor-infiltrating cytotoxic T cells

**Fig. 7:**
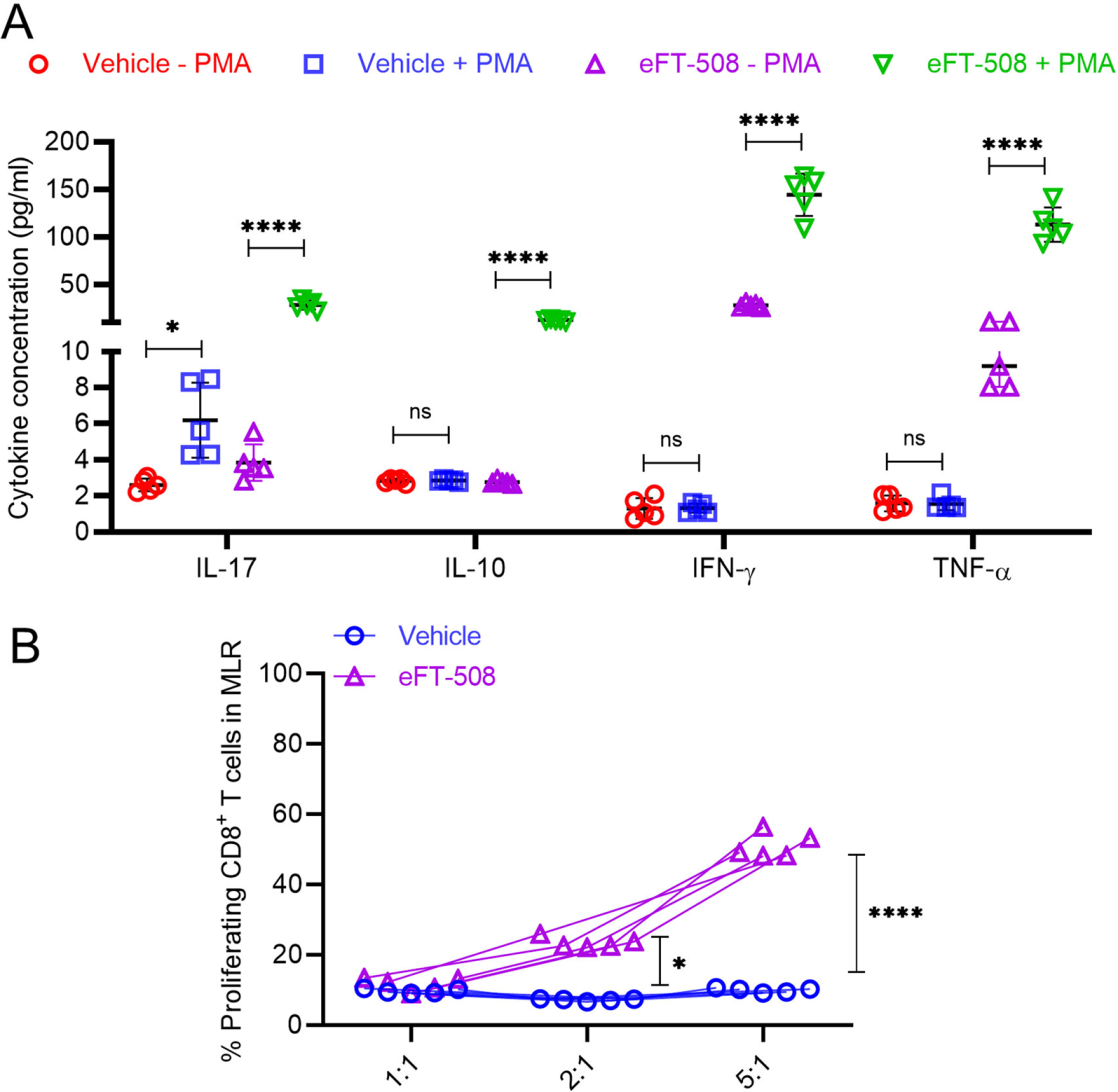
Treatment with eFT-508 restores the functional activity of tumor-infiltrating CD8 + T cells. **A** Expression of cytokines in tumor-infiltrating CD8 + T cells isolated from mice treated with vehicle control or eFT-508 ± PMA/Ionomycin. Data are mean ± SD. **B** Effector function was evaluated by a mixed lymphocyte reaction (MLR) assay. CD8 + T cells isolated from vehicle-treated mice were dysfunctional in comparison to cells isolated from eFT-508 treated mice (n = 5). *P < 0.05, ****P < 0.0001, ns—not significant (*P* > 0.05)

Since the overexpression of PD-L1 was associated with tumor exhaustion and functional impairment [20, 21], we hypothesized that eFT-508-mediated inhibition of PD-L1 expression could restore the effector function of tumor-infiltrating CD8 + T cells (TILs). Hence, the functional capacity of tumor-infiltrating CD8 + T cells in mice treated with vehicle and eFT-508 monotherapy was assessed. CD8 + T cells isolated from mice treated with vehicle demonstrated attenuated expression of Th1/Th2 cytokines, interleukin (IL)-17, interleukin (IL)-10 interferon-gamma (IFN- γ), and tumor necrosis factor-alpha (TNFα), both in the native state and after polyclonal stimulation with PMA and ionomycin (Fig. 7A). In comparison, the expression of all four cytokines was higher in tumor-infiltrating T cells isolated from mice treated with eFT-508 in the native state and significantly induced after polyclonal stimulation (Fig. 7A). Furthermore, the effector function of tumor-infiltrating CD8 + T was determined using the mixed lymphocyte reaction (MLR) assay. The tumor-infiltrating CD8 + T cells in mice treated with vehicle control had impaired alloreactivity compared to those in mice treated with eFT-508 (Fig. 7B), consistent with the altered effector function. Thus, these results established that treatment with eFT-508 alone or in combination with adriamycin had a synergistic effect on tumor growth, metastatic disease progression, potentially due to its ability to restore effector function of tumor-infiltrating cytotoxic CD8 + T cells.

## Discussion

Patients with TNBC responded better to neoadjuvant chemotherapy; however, OS and PFS still decreased [11, 22]. Therefore, there was an urgent need to find alternative treatment strategies. The evasion of immune surveillance is a crucial adaptive mechanism for tumor progression [23]. The expression of immune checkpoint receptors by tumor-infiltrating T cells and their cognate ligands by tumor cells is a potent mechanism to drive T cell exhaustion, resulting in impaired T cell function and immune evasion [20, 21]. In this study, we found that eFT-508, an MNK1/2 inhibitor, enhanced the sensitivity of in vitro chemotherapy and prevented tumor growth and metastasis in vivo by inhibiting the expression of PD-L1. Our results indicated that eFT-508 mediated its anti-tumorigenic activity partially by inhibiting PD-L1 expression and restoring the effector function of tumor-infiltrating cytotoxic T cells. Of note, MDA-MB-231 cells were more chemosensitive compared to the MDA-MB-468 cells, despite both of them being TNBC cell lines. This is potentially due to the fact that MDA-MB-468 is a basal BC cell line whereas MDA-MB-231 is a claudin-low cell line. MDA-MB-231 is characterized by low expression of claudin 3, 4, and 7, as well as low expression of Ki67 and the epithelial cell marker E-cadherin. In contrast expression of each one of these markers is higher in the MDA-MB-468 cells. So, the observed difference among these two TNBC cell lines can be due to relative enrichment of mesenchymal cell markers and cancer stem cells [24–26].

PD-L1/PD1 is a key molecule in tumor development. Despite significant improvements in PD-L1-PD-1 blockade in different cancers, only a fraction of patients have been found to show durable and objective clinical response among the high incidence of intrinsic and acquired therapy resistance [24]. Thus, it was essential to understand how the expression of PD-L1 was controlled in chronic conditions to tackle these challenges. PD-L1 has been shown to be regulated at genetic, epigenetic, transcriptional, translational, and posttranslational levels [27–37], often in a context-dependent fashion. Therefore, we targeted PD-L1 to detect the effect of drugs on tumors. Additionally, PI3K/Akt activation, MAPK signaling, as well as transforming growth factor-beta (TGFβ) and tumor necrosis factor-alpha (TNFα) are known oncogenic signals that can induce PD-L1 expression [27, 31–33, 38, 39]. Indeed, PD-L1 expression is known to be inversely regulated by the genetic ablation of PTEN in patients with TNBC [39]. MicroRNA (miR)-200 has been shown to inhibit translation of *CD274* mRNA (which encodes PD-L1) [32, 36]. The miR-200 family is known to be regulated by TGFβ signaling pathway and functions as an inhibitor of EMT and metastatic progression in BC [12, 13]. Thus, the loss of miR-200 expression could induce the expression of PD-L1, in turn helping tumors to evade immune surveillance. Additionally, more studies are required to understand how PD-L1 is regulated and whether eFT-508 regulates tumor immunity through PD-L1.

eFT-508 is known to affect tumor growth. Studies have found that eFT-508 reduces the development and progression of liver cancer in myc-driven mouse model [17]. However, the therapeutic effect of eFT-508 on other tumors still needs further exploration. Cancer cells have evolved various cellular mechanisms for stress-adaptive translation [16, 17, 40–42]; hence, therapeutic targeting of translation has evolved as a selective yet efficacious regimen [16]. PD-L1 inhibitors are partially effective, and PD-L1 transcripts are independent of the overall survival of patients with BC. Therefore, eFT-508 contributed to the partial effectiveness of PD1/PD-L1 inhibitors. Targeting these specialized adaptive translation mechanisms might dramatically improve the therapeutic index.

## Conclusions

Whether eFT-508 regulated the expression of additional ligands of immune checkpoint receptors was unclear. EFT-508 seems to cause global translation inhibition. Hence, it was unclear whether the action of eFT-508 on PD-L1 expression was a direct effect or mediated indirectly by other factor(s). Furthermore, dose- and duration-based effect on toxicity and durability of antitumor response of eFT-508 in the in vivo models of TNBC remains to be determined. A phase 2, open-label study (ClinicalTrials.gov Identifier: NCT03616834) is currently being run by Effector Therapeutics to evaluate the safety profile, pharmacokinetics, and durability of response in patients who were already on anti-PD-1/anti-PD-L1 monotherapy but had progressive disease or had undergone 12 weeks of treatment with no evidence of complete or partial response. Based on our findings in the current study that eFT-508 could inhibit PD-L1 expression in TNBC and similar evidence in liver cancer [17], we speculated that the ongoing clinical trial could establish clinical synergism. Finally, whether eFT-508 had a similar effect on disease outcome in other classes of BC or if it was specific to TNBC needs further investigation.

## Supplementary Information

**Additional file 1: Figure S1.** Expression of CD274 transcript is not correlated to disease stage or race. (A) Boxplots showing the expression of CD274 mRNA in normal breast tissue and BC of Stage 1 to 4 (UALCAN). (B) Boxplots showing the expression of CD274 mRNA in normal breast tissue and BC tissues from patients of the indicated race (UALCAN).

## Data Availability

All data generated or analyzed during this study are included in this published article and its Additional information files.
