# Supplementary figures and images for: Treatment with eFT-508 increases chemosensitivity in breast cancer cells by modulating the tumor microenvironment
Source: J Transl Med. 2022 Jun 18;20:276. doi: 10.1186/s12967-022-03474-9 (PMC9206753; doi:10.1186/s12967-022-03474-9)

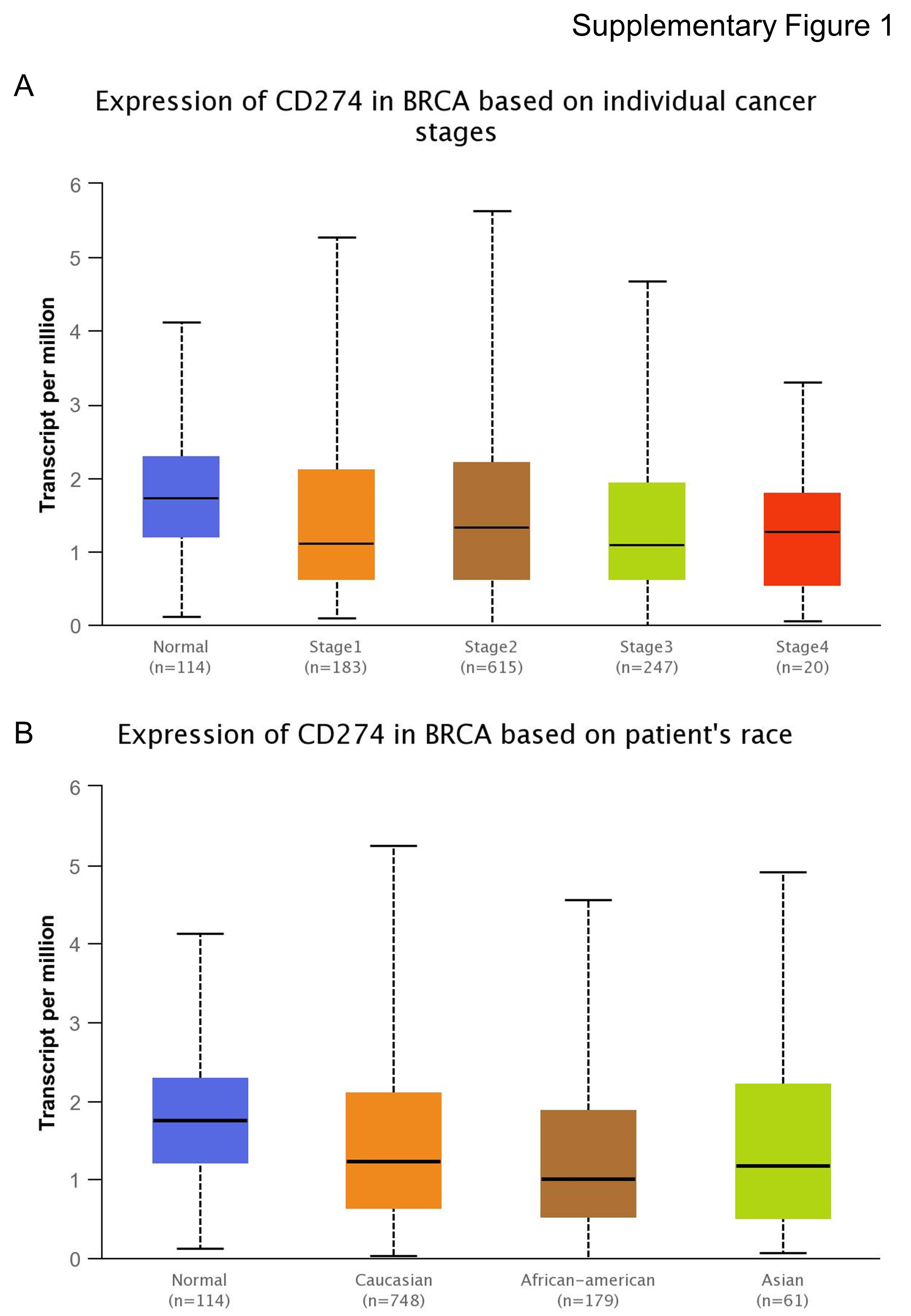

Supplement: Supplementary file 1 — Additional file 1: Figure S1. Expression of CD274 transcript is not correlated to disease stage or race. (A) Boxplots showing the expression of CD274 mRNA in normal breast tissue and BC of Stage 1 to 4 (UALCAN). (B) Boxplots showing the expression of CD274 mRNA in normal breast tissue and BC tissues from patients of the indicated race (UALCAN). [file 12967_2022_3474_MOESM1_ESM.jpg]
